# A Subset of Patients with Acute Myeloid Leukemia Has Leukemia Cells Characterized by Chemokine Responsiveness and Altered Expression of Transcriptional as well as Angiogenic Regulators

**DOI:** 10.3389/fimmu.2016.00205

**Published:** 2016-05-25

**Authors:** Annette K. Brenner, Håkon Reikvam, Øystein Bruserud

**Affiliations:** ^1^Section for Hematology, Department of Clinical Science, University of Bergen, Bergen, Norway; ^2^Department of Medicine, Haukeland University Hospital, Bergen, Norway

**Keywords:** acute myeloid leukemia, chemokine, CCL28, integrin, RNA, epigenetic, helicase

## Abstract

Acute myeloid leukemia (AML) is an aggressive and heterogeneous bone marrow malignancy, the only curative treatment being intensive chemotherapy eventually in combination with allogeneic stem cell transplantation. Both the AML and their neighboring stromal cells show constitutive chemokine release, but chemokines seem to function as regulators of AML cell proliferation only for a subset of patients. Chemokine targeting is therefore considered not only for immunosuppression in allotransplanted patients but also as a possible antileukemic strategy in combination with intensive chemotherapy or as part of disease-stabilizing treatment at least for the subset of patients with chemokine-responsive AML cells. In this study, we characterized more in detail the leukemia cell phenotype of the chemokine-responsive patients. We investigated primary AML cells derived from 79 unselected patients. Standardized *in vitro* suspension cultures were used to investigate AML cell proliferation, and global gene expression profiles were compared for chemokine responders and non-responders identified through the proliferation assays. CCL28-induced growth modulation was used as marker of chemokine responsiveness, and 38 patients were then classified as chemokine-responsive. The effects of exogenous CCL28 (growth inhibition/enhancement/no effect) thus differed among patients and was also dependent on the presence of exogenous hematopoietic growth factors as well as constitutive AML cell cytokine release. The effect of CCR1 inhibition in the presence of chemokine-secreting mesenchymal stem cells also differed among patients. Chemokine-responsive AML cells showed altered expression of genes important for (i) epigenetic transcriptional regulation, particularly lysine acetylation; (ii) helicase activity, especially DExD/H RNA helicases; and (iii) angioregulatory proteins important for integrin binding. Thus, chemokine responsiveness is part of a complex AML cell phenotype with regard to extracellular communication and transcriptional regulation. Chemokine targeting in chemokine-responsive patients may thereby alter AML cell trafficking and increase their susceptibility toward antileukemic treatment, e.g., conventional chemotherapy or targeting of other phenotypic characteristics of the chemokine-responsive cells.

## Introduction

Acute myeloid leukemia (AML) is an aggressive disease characterized by infiltration of malignant myeloblasts in the bone marrow; the only curative treatment is intensive chemotherapy potentially in combination with allogeneic stem cell transplantation (1). However, such intensive treatment is not applicable for elderly or unfit younger patients due to an unacceptable risk of severe toxicity and treatment-related mortality; immunological complications are important causes of the transplant-related mortality that can be due to acute graft vs. host disease (GVHD) as well as severe infections due to the immunocompromised state of patients with chronic GVHD (1).

Several strategies for targeted therapy in AML are now considered (2, 3). One possibility is to target the AML-supporting communication between AML cells and their neighboring stromal cells that mediate growth support through their release of soluble mediators, including several chemokines (4–11). Chemokines are involved in this communication; they may function as growth regulators or be important for keeping the leukemic cells in their permissive microenvironment and thereby render them less susceptible to antileukemic therapy. However, *in vitro* studies suggest that chemokines function as growth regulators in leukemic hematopoiesis only for a subset of AML patients, and a wide range of both CCL and CXCL chemokines can then modulate leukemia cell proliferation (4). One of these chemokines is CCL28 (4) that is released by non-leukemic bone marrow stromal cells, and that preserves the functional integrity of normal hematopoietic progenitor cells (12) through binding to the G-protein-coupled receptors (GPCRs) CCR3 and CCR10 (13–15). CCR3 is a promiscuous receptor, which can bind several ligands in addition to CCL28, whereas CCR10 can only bind CCL27 and CCL28 (16).

Our previous studies have identified a subset of patients whose AML cells show altered proliferation in the presence of exogenous chemokines, and the aim of the present study was to give a broader and more detailed characterization of the AML cell phenotype for these chemokine-responsive patients. First, chemokine-responsive patients show growth modulation in the presence of several chemokines, including CCL28. We therefore used CCL28 responsiveness to identify the chemokine-responsive subset among 79 unselected patients, and because CCL28 is important in normal hematopoiesis, we in addition wanted to characterize both the effects of exogenous CCL28 and chemokine receptor inhibition in leukemic hematopoiesis as parts of our phenotype studies. Second, the phenotype of the chemokine-responsive patient subset was further characterized by comparison of global gene expression profiles for chemokine-responsive and non-responsive patients. A more detailed characterization of this phenotype would be necessary in order to design clinical studies and decide optimal clinical use of targeted therapy in this subset of AML patients.

## Materials and Methods

### AML Patients and AML Cell Preparation

The study was conducted in accordance with the Declaration of Helsinki, and the protocol was approved by the local Ethics Committee (Regional Ethics Committee III, University of Bergen). Samples were collected after written informed consent. AML blasts were derived from 79 consecutive patients (34 females and 45 males; median age 67 years with range 18–87 years). Six patients had AML relapse (Table 1) and 11 patients had acquired AML secondary to previous hematological disease (10 patients) or chemotherapy (1 patient). Cytogenetic analyses were available for 71 patients; 9 patients had favorable, 6 patients intermediate, 15 patients adverse, and 41 patients normal cytogenetics, respectively. Our selection of patients and the methods for preparation (gradient separation alone) and characterization of AML cells have been described in detail previously (17).

**Table 1:** **Clinical and biological characteristics of the 79 unselected patients admitted to our hospital for AML treatment and included in the present study**.

Patient characteristics		Cell morphology		Cell genetics	
Age		FAB classification		Cytogenetics^a^	
Median (years)	67	M0	6	Favorable	9
Range (years)	18–87	M1	21	Intermediate	6
		M2	11	Normal	41
Gender		M3	2	Adverse	15
Females	34	M4	18	n.d.	8
Males	45	M5	15		
		n.d.	6	Flt3 mutations	
Secondary AML				ITD^b^	28
MDS	7	CD34 receptor		Wild-type	37
Chemotherapy	1	Negative (≤20%)	21	n.d.	14
CM(M)L	3	Positive (>20%)	53		
		n.d.	5	NPM1 mutations	
AML relapse	6			Mutated	26
				Wild-type	40
				n.d.	13

*^a^One of the patients had cytogenetic abnormalities with different prognostic impact; inv(16) is associated with a favorable prognosis and +8 with an adverse prognosis. Inv(16) is regarded to have the strongest impact and therefore to neutralize the negative impact of +8, the patient was classified as having a favorable karyotype*.

*^b^One of the patients has an additional point mutation at D835*.

*n.d., not determined*.

Prior to the study, we decided to include 80 unselected patients (one patient was left out due to technical reasons); based on a previous study (4), we would then expect to identify at least 25 chemokine responders. This number would be sufficient for statistical comparisons of functional characteristics as well as global gene expression profiles.

The study included only patients with a high percentage (>80%) and a high absolute number (>15 × 10^9^/L) of leukemia cells among the peripheral blood leukocytes (18). A cell population, including >95% AML cells, could then be prepared by density gradient separation alone (19). Cells were cryopreserved and stored in liquid nitrogen until used in the experiments (19).

### Functional *In Vitro* Characterization of Primary Human AML Cells

All cultures of AML cell alone were prepared in serum-free medium (Stem Span, Stem Cell Technologies, Vancouver, BC, Canada), and all recombinant cytokines were supplied by PeproTech (Rocky Hill, NJ, USA). All exogenous cytokines were added at 20 ng/mL, i.e., corresponding to an excess of the added cytokine. Our methods for flow-cytometric characterization of AML cell viability (20), spontaneous and cytokine-dependent proliferation in suspension cultures determined by ^3^H-thymidine incorporation (4, 17), constitutive cytokine release (4), and analysis of AML cell viability and proliferation (^3^H-thymidine incorporation) in transwell cocultures with bone marrow mesenchymal stromal cells [(MSC); MSC24539 purchased from Lonza, Cambrex BioScience, Walkersville, MD, USA] (8, 9) have been described in detail previously. CCL28 levels were determined by enzyme-linked immunosorbent assay (ELISA) analysis (R&D Systems, Abingdon, UK), the minimal detectable level being 45 pg/mL. CXCL2 levels were also determined by ELISA analysis, whereas the levels of the other chemokines were investigated by Luminex analyses (R&D Systems). The combined CCR1 and CCR3 antagonist J113863 (R&D Systems) was used at a final concentration of 1.5 μM for chemokine receptor inhibition.

### RNA Preparation and Analysis of Global Gene Expression

All microarray data were performed using the Illumina iScan Reader, which is based upon fluorescent detection of biotin-labeled cRNA; 300 ng total RNA from each sample was reversibly transcribed, amplified, and labeled with Biotin-16-UTP using the Illumina TotalPrep RNA Amplification Kit (Applied Biosystems/Ambion, Foster City, CA, USA). Amount and quality of the biotin-labeled cRNA were controlled both by NanoDrop spectrophotometer and Agilent 2100 Bioanalyzer, before 750 ng of biotin-labeled cRNA was hybridized to the HumanHT-12 V4 Expression BeadChip according to the manufacturer’s instructions. The chip targets 47,231 probes derived primarily from genes in the NCBI RefSeq database (Release 38).

### Bioinformatical and Statistical Analyses

Bioinformatical analyses were performed using the J-Express 2012 software (MolMine AS, Bergen, Norway). For hierarchical clustering of the cytokine secretion, all values were median-normalized and log(10)-transformed. Complete linkage and Pearson correlation were used as linkage method and distance measurement, respectively. The statistical analyses were performed with the IBM Statistical Package for the Social Sciences (SPSS) version 23 (Chicago, IL, USA). The Mann–Whitney *U*-test was used to compare value distributions among different patient groups, and χ^2^ tests (Pearson’s χ^2^ test and the likelihood ratio) were used to determine correlations between different categories. Additionally, Kendall’s tau-*b* correlation tests were performed to determine the agreement between cytokine expression levels in cell supernatants and the mRNA levels of these cytokines. *P*-values <0.05 were regarded as statistically significant.

## Results

### Identification of a Chemokine-Responsive Patient Subset: Exogenous CCL28 Does Not Alter Spontaneous *In Vitro* Apoptosis but Modulates AML Cell Proliferation for a Subset of Patients

There was a wide variation among the 79 AML patients with regard to the viability of the leukemic cells after 40 h of *in vitro* culture in medium alone (median viability 36%, range 2–81%). The presence of 20 ng/mL exogenous CCL28 during culture did not significantly alter AML cell viability (median viability 35%, range 3–78%) when comparing the overall results (data not shown). Thus, exogenous CCL28 does not have any major effect on the regulation of spontaneous *in vitro* apoptosis for primary human AML cells.

Proliferation was analyzed using the ^3^H-thymidine incorporation assay, where the nuclide was added after 6 days and nuclear activity assayed 24 h later. A median value of at least 1,000 counts per minute (cpm) in triplicate cultures was defined as *detectable proliferation*, whereas a *significant alteration* of ^3^H-thymidine incorporation was defined as (i) an increase/decrease corresponding to at least 20% of the control culture, and the absolute value of this change being at least 2,000 cpm for patients with detectable proliferation or alternatively, (ii) a change from detectable to undetectable proliferation or *vice versa* (4, 17). We first compared AML cell proliferation for cultures prepared in medium alone and medium supplemented with 20 ng/mL of CCL28 for all 79 patients. The proliferation in medium alone varied among the 79 patients (range <1,000–9,191 cpm), and only 17 patients showed detectable autocrine (i.e., spontaneous) proliferation. Exogenous CCL28 increased the proliferation for 14 of the 79 patients compared with the corresponding control cultures prepared in medium alone, whereas decreased proliferation was seen for 3 patients.

### The Chemokine-Responsive AML Cell Phenotype: CCL28-Associated Growth Modulation Depends On the Local Cytokine Network

**Figure 1:**
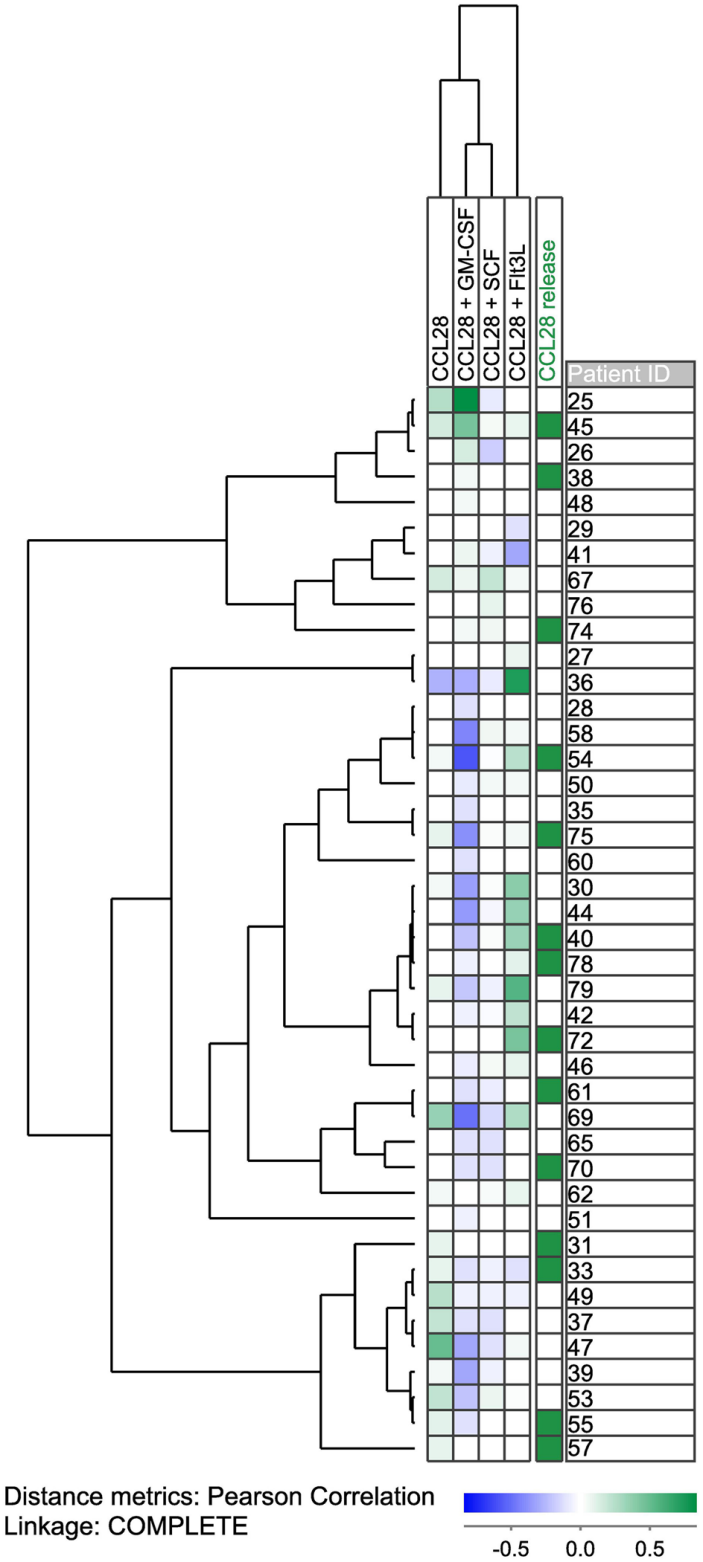
**CCL28-associated growth modulation in primary human AML cells; hierarchical clustering analysis of the effects of exogenous CCL28 on the spontaneous and GM-CSF/SCF/Flt3L-dependent proliferation of AML blasts derived from 56 unselected patients**. AML cells were cultured in medium alone or medium supplemented with exogenous GM-CSF, SCF, or Flt3L; for all four types of culture, we compared proliferation for cells cultured with and without exogenous CCL28. All cytokines were tested at a final concentration of 20 ng/mL. For 14 patients, undetectable proliferation (corresponding to a median incorporation of <1,000 cpm for triplicate determinations) was detected for all cultures; for this reason, we only present the results for the remaining 42 patients. The leukemic cells were cultured in suspension cultures, and the proliferation was measured as ^3^H-thymidine incorporation after 7 days of culture.

We investigated the effect of CCL28 on growth factor-dependent proliferation of primary human AML cells for 56 patients that represent a consecutive and thereby unselected subset of the 79 patients examined in the autocrine proliferation studies described above. The effect of CCL28 was tested in suspension cultures prepared either in medium alone or medium supplemented with exogenous granulocyte macrophage colony-stimulating factor (GM-CSF), stem cell factor (SCF), or FMS-like tyrosine kinase 3 ligand (Flt3L). A detectable proliferative response for at least one of these three growth factors was detected for 42 of the 56 patients, and the results for these patients are summarized in the hierarchical cluster analysis presented in Figure 1 (the analysis also includes the effect of CCL28 for cells cultured in medium alone, see above). We defined a significant alteration as a difference between the CCL28-containing and the corresponding control culture as either (i) a change from/to undetectable levels or (ii) a difference corresponding to an absolute value of at least 2,000 cpm and in addition corresponding to >20% of the respective control culture. It can be seen that exogenous CCL28 had divergent effects on AML cell proliferation depending on the local cytokine network. When using these definitions, we observed a significant growth inhibition for 21 and a growth enhancement for 3 out of the 42 patients in the presence of GM-CSF. In contrast, in the presence of Flt3L, significant growth enhancement was seen for 11 patients, and growth inhibition was observed for 3 patients. Finally, CCL28 had generally weaker effects in the presence of SCF; significant inhibition was seen only for six patients and enhancement for two patients. To summarize, CCL28 can alter the proliferation of primary human AML cells for a considerable number of patients, this effect is highly dependent on the local cytokine network and the presence of hematopoietic growth factors, and exogenous CCL28 altered either autocrine or cytokine-dependent (GM-CSF, Flt3L, and SCF) proliferation for 36 of the 56 patients.

CCL28 can bind to the CCR3 and CCR10 receptors (13–15). We used our global gene expression profiles (see below) to compare the expression of CCR3 and CCR10, but we could not detect any significant difference in receptor expression between patients with and without CCL28-associated growth modulation (data not shown). These observations suggest that the differences in chemokine responsiveness between patients is not caused by different regulation of CCR3/CCR10 expression at the mRNA level.

### Additional Phenotypic Characteristics of Chemokine-Responsive AML Cells: Constitutive CCL28 Release by AML Cells Is Detected for a Minority of Patients, Showing Weak Association with the Release of Other Chemokines and No Association with Chemokine Responsiveness

The constitutive release of 11 chemokines was investigated for all 79 patients (2 × 10^6^ cells/mL, 48 h of *in vitro* culture). Detectable CCL28 release was observed for only 18 patients (median supernatant level 126 pg/mL, range 49–812 pg/mL), whereas the other chemokines were released for most patients (Table 2). When comparing the overall results, no significant correlation was seen between detectable CCL28 release and cell viability (i.e., spontaneous *in vitro* apoptosis), differentiation [morphological signs according to French–American–British (FAB) classification, expression of the CD34 stem cell marker], karyotype, and *Flt3* or *NPM1* mutations (data not shown). Finally, there was no significant association between constitutive CCL28 release and spontaneous/autocrine *in vitro* AML cell proliferation, and constitutive CCL28 release was seen both for patients with and without CCL28-associated growth modulation.

**Table 2:** **Constitutive chemokine release by primary human AML cells; a summary of the results for the 79 unselected patients included in the present study**.

Chemokine	# patients with detectable release	Median conc. (pg/mL)	Range (pg/mL)
CCL2	67	110	n.d.–9,807
CCL3	79	200	119 to >30,000
CCL4	79	92	21–11,744
CCL5	79	39	7.4–2,481
*CCL28*	*18*	*n.d*.	*n.d*.–*812*
CXCL1	79	79	41–22,772
CXCL2	69	16	n.d.–11,594
CXCL5	78	95	n.d. to >14,500
CXCL8	79	535	0.3 to >18,500
CXCL10	79	12	1.4–24,642
CXCL11	79	83	25–246

*The levels were determined in the culture supernatants after 48 h of *in vitro* culture for the primary AML cells, the supernatant levels of 11 chemokines were determined, and the results are presented as the number of patients with detectable release, the median level for all 79 patients, and the variation range. CCL28 is highlighted*.

*n.d., not detected, i.e., below detection limit*.

**Figure 2:**
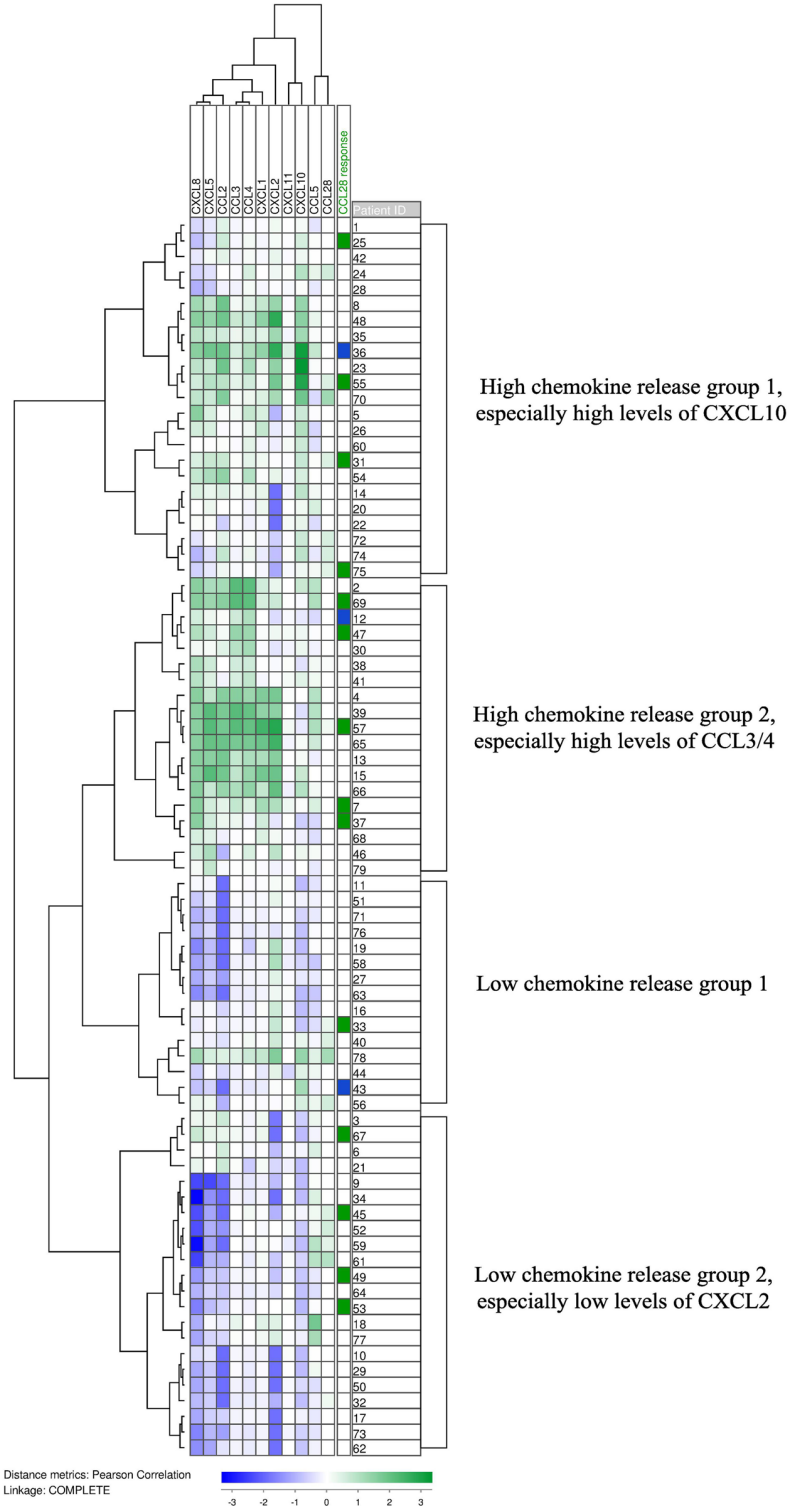
**The constitutive chemokine release profile by primary human AML cells**. The constitutive release of 11 chemokines, including CCL28, during *in vitro* culture was examined for primary human AML cells derived from 79 consecutive patients. Chemokine release was determined as the protein level in culture supernatants. Based on the hierarchical clustering analysis, the patients could be divided into four subclusters. CCL28, together with CCL5, formed an out-group and did not correlate with the expression of the other nine chemokines. The column on the right shows the 14 patients whose cells responded with increased proliferation to CCL28 (green) in medium alone without supplementation of exogenous growth factors, and the 3 patients whose blasts showed reduced proliferation in the presence of CCL28 compared to cultures containing only medium (blue).

We have previously investigated the constitutive chemokine release for a consecutive patient group, and in the present study, we also determined the supernatant levels for 10 additional chemokines commonly released by primary human AML cells at relatively high levels (79 patients). The chemokine levels for each individual patient was median-normalized and log(10)-transformed prior to Pearson clustering in J-Express. The analysis is presented in Figure 2. Several chemokines were then grouped together in defined subsets. Our previous studies have shown that there are three different chemokine subsets characterized by close clustering and significant correlations between supernatant levels (4); this was also confirmed by our present clustering analysis of constitutive chemokine release: (i) CXCL10/11 clustered close to CCL5; (ii) CCL2/CXCL1/5/8 clustered close to each other; whereas (iii) CXCL2 was the only chemokine tested out of the five members of the third cluster and did not cluster close to CCL28 (4). CCL28 as well as CCL5 clustered as outliers without any strong association with other members of the main chemokine clusters.

It can be seen from Figure 2 that the patients could be divided into four main subsets/clusters; the two upper subsets generally showed high constitutive chemokine release. CCL28-induced growth inhibition for cultures prepared with GM-CSF and SCF was significantly more frequent in this high chemokine release group (15 and 6 out of the upper 42 patients, respectively) compared with the low-release group (6/37 and 0/37; likelihood ratios = 0.023 and 0.003, respectively). This observation further supports our hypothesis from the studies of CCL28 effects in the presence of exogenous cytokines (GM-CSF, Flt3L, and SCF, see above); the final effects of CCL28 on AML cell proliferation depends on the local cytokine network determined by the constitutive cytokine release by the leukemic cells (Figure 2) and exogenous cytokines (i.e., cytokines not released by AML cells).

### Additional Phenotypic Characterization of AML Cells: Normal Blood Cell Counts at Diagnosis Do Not Differ among Patients with and without Constitutive CCL28 Release

Bone marrow failure with pancytopenia in peripheral blood is common at the time of diagnosis for AML patients (1). Furthermore, CCL28 is a growth and survival factor for normal hematopoietic cells (12), and for this reason we compared peripheral blood hemoglobin levels and platelet counts at the time of diagnosis for patients with and without constitutive CCL28 release by their AML cells. We could not detect any significant difference between the two groups with regard to Hb levels or platelet counts at the time of diagnosis (data not shown).

### Additional Phenotypic Characterization of Chemokine-Responsive Patients: Chemokine Receptor Blocking Has Antileukemic Effects Only for Certain Patients

We investigated the effects of endogenous chemokines using an *in vitro* model, where primary human AML cells and normal bone marrow MSCs derived from a healthy donor were separated by a semipermeable membrane during coculture (9); the MSCs can then influence AML cell proliferation only through their release of soluble mediators. We tested the effects of the combined CCR1 (ligands being CCL2/3/5/7/14/15/16/23/28) and CCR3 (ligands being CCL5/7/8/11/13/15/24/28) antagonist J113863 on AML cell viability and proliferation; viability was investigated by a flow-cytometric assay and proliferation as ^3^H-thymidine incorporation (8, 9). We investigated this pharmacological effect for primary AML cells derived from five CCL28/chemokine responders. The inhibitor had divergent effects on AML cell proliferation during coculture with chemokine-releasing MSCs; for one patient, growth inhibition was seen (median proliferation corresponding to 9,457 vs. 3,502 cpm), for three other patients, increased proliferation was observed (21,323 vs. 26,253 cpm, 10,934 vs. 19,713 cpm, and 55,257 vs. 74,956 cpm, respectively), and for the last patient, only a minor effect of CCL28 was seen (4,800 vs. 4,392 cpm) in the cocultures. These experiments show that chemokines mediate divergent effects on AML cell proliferation that differs among the chemokine responders also, when tested in the presence of cytokine/chemokine-releasing bone marrow MSCs.

### Additional Phenotypic Characterization of Chemokine-Responsive Patients: Chemokine Responsiveness Is Associated with Altered Expression of Genes Important for Protein Acetylation, Helicase Activity, and Integrin Binding

In a previous study, we observed that CCL28 could modulate AML cell proliferation only for a subset of patients (4); this was also confirmed in our present study (Figure 1). The previous study showed that for this particular patient subset, AML cell proliferation could be modulated not only by CCL28 but also by several other chemokines binding to different chemokine receptors (4). Thus, CCL28 growth modulation should be regarded as a marker of a patient subset that, in contrast to other patients, are characterized by chemokine-induced (GPCR-mediated) growth modulation. We then compared the global gene expression profiles for 37 unselected patients; 15 of these patients being classified as responders, according to the definitions used above for the effects of exogenous CCL28 on spontaneous and/or growth factor-dependent (GM-CSF, Flt3L, and SCF) proliferation. However, with regard to the classification of patients as responders/non-responders and the change to/from undetectable from/to detectable proliferation (i.e., proliferation corresponding to >1,000 cpm defined as detectable, see above), this definition is arbitrary and four of the seven patients with such a change showed a relatively small absolute difference between cultures with and without exogenous CCL28. Even though there was a change from undetectable to detectable with CCL28, two of these four patients therefore were by chance classified as responders and the two others as non-responders.

We first identified those genes showing a difference between the two patient subsets corresponding to *p* < 0.001 and a false discovery rate (FDR) of <0.01. The identified genes (21) were included in 20 gene ontology (GO)-terms; a major part of these terms were associated with integrin binding (Table 3, Table S1 in Supplementary Material), protein/lysine/histone acetylation (Table 4, Table S2 in Supplementary Material), or helicase activity (Table 5, Table S3 in Supplementary Material). The term integrin binding included 27 differentially expressed genes; among them were several proteases and angioregulatory mediators, seven genes encoding extracellular matrix molecules, and several adhesion molecules. The large part of the proteins encoded by these genes seems to play a role in the regulation of local angiogenesis, a process important both for the development and chemosensitivity of human AML (8). Integrins may also be important for modulation of intracellular signaling initiated by ligation of the CCR3 chemokine receptor (22). Second, the terms histone acetylation, lysine-protein acetylation, and internal peptidyl lysine acetylation were overlapping and included 48 genes; a large number of them are involved in histone modulation/acetylation and/or in transcriptional regulation (Table 4, Table S2 in Supplementary Material). Finally, the largest group of differentially regulated genes were included in the GO term Helicase Activity (Table 5, Table S3 in Supplementary Material). This group included mainly RNA helicases (e.g., 23 members of the DEAD box family RNA helicases together with several DEAH box family RNA helicases), mini-chromosome maintenance proteins, and members of the sucrose non-fermentable/SWItch (SNF/SW1) family.

**Table 3:** **Comparison of global gene expression profiles for primary human AML cells with and without chemokine-induced growth modulation – a summary of the differentially expressed genes encoding proteins important for integrin binding (for additional details, see Table S1 in Supplementary Material)**.

Classification of genes based on the protein function	Angioregulation vascular biology
**Proteases**	
ADAM2, a disintegrin and metallopeptidase (ADAM) domain 2	+
ADAM23, ADAM metallopeptidase domain 23	+
ADAM22, ADAM metallopeptidae domain 22	+
ADAMTS8, ADAM metallopeptidase with thrombospondin type 1 motif 8	+
**Extracellular matrix molecules**	
COL3A1, collagen type III alpha 1	+
COL5A1, collagen type V alpha 1 (vascular stability)	
COL16A1, collagen type XVI alpha 1	
FBLN5, fibulin 5	+
LAMA5, laminin alpha 5	+
LAMB2, laminin beta 2 (angioregulator)	+
TNN, tenascin N	+
**Soluble mediators involved in angiogenesis**	
ANGPTL1, angiopoietin-like 1	+
CYR61, cysteine-rich angiogenic inducer 61	+
JAM3, junctional adhesion molecule 3 (the soluble form)	
VWF, von Willebrand factor	+
**Cell-to-cell and cell-to-matrix adhesion**	
ICAM3, intercellular adhesion molecule 3 (LFA-1 ligand)	+
ITGB1BP1, integrin beta 1 binding protein 1	+
ITGB6, integrin beta 6	+
JAM3, junctional adhesion molecule 3	+
THBS4, thrombospondin 4	+
THY1, Thy-1 cell surface antigen	+
**Other genes encoding proteins involved in angiogenes**	
EMP2, epithelial membrane protein 2 (integrin modulator)	+
KDR, kinase insert domain receptor (VEGF receptor)	+
NMB (neuromedin B)	+
SOD1, superoxide dismutase 1, soluble	+
**Other genes**	
IMPAD1, inositole monophosphatase domain containing 1	
OXCT1, 3-oxoacid transferase 1 (mitchondrial metabolism)	

**Table 4:** **A comparison of the global gene expression profiling of primary human AML cells with and without chemokine-induced growth modulation – a summary of the differentially expressed genes that encode proteins important for histone acetylation (for additional details, see Table S2 in Supplementary Material)**.

Main classification based on the protein function
Gene identity
**Chromatin modulation/histone acetylation and transcriptional regulation**
ACTL6A, CHD9, DMAP1, EPC1, ING3, KAT2A, KAT2B, KIAA1267, MEAF6, MLL, MYST1, MYST2, MYST4, SRCAP, SMARCA4, TRRAP, YEATS2
**Transcriptional regulation only**
BRD8, BRPF1, CHD9, GTF3C4, EP400, HCFC1, LDB1, MECP2, MYOD1, OGT, PHD15, PHD16, PHF17, PHF20, TAF1, TAF1L, TAF6L, TAF15, TCF3
**DNA repair**
BRCA2, HCFC1, PHF20, TRRAP
**Cell cycle regulation**
HCFC20
**Genes with other or unknown functions**
BAT3 (apoptosis), CCDC101, CPA3 (protease), KIAA1310, MBIP, MSL2, USP22

**Table 5:** **Comparison of global gene expression profiles of primary human AML cells with and without chemokine-induced growth modulation – a summary of the differentially expressed genes encoding proteins important for helicase activity**.

Classification of genes based on the protein structure and/or function	DNA	RNA	Trans.
**DEAD box family of proteins (RNA helicases)**			
DDX4, DDX10 (involved in leukemogenesis?), DDX17, DDX18 (activated by Myc)		**+**	
DDX27/50/51/52/54/58/60/60L			
DOX19A		**+**	
IFIH1		**+**	
DDX3X/6/28		**+**	**+**
**DEAH box family of proteins**			
DHX8/9/40		**+**	
DHX29			
WRN (DNA repair?)	**+**		
**Mini-chromosome maintenance proteins (genome replication)**			
MCMDC1 (chromatin modulation)			
MCM7/9			
MCM8	**+**		
**SNF/SW1 family of protein (chromatin remodeling)**			
ATRX			**+**
HLTF			**+**
SMARCA2, SMARCA4, SMARCAL1			**+**
**Other helicases**			
CHD3 (histone deacetylation)			**+**
CHD4 (histone deacetylation)	**+**		**+**
CHD6 (chromatin remodeling)			**+**
CHD8 (chromatin remodeling)	**+**		**+**
CHD9	**+**		
MOV10		**+**	
HELZ		**+**	
**Proteins involved in DNA methylation, chromatin remodeling, and DNA repair**			
ASCC3 (DNA repair)			
ERCC8 (DNA repair), SHPRH (DNA repair?), SRCAP (histone remodeling)			**+**
**Other genes/proteins**			
EIF4A1			
EP400 (protein synthesis?)			
GTF2F2			**+**
JARID2 (mutations associated with myeloid malignancies)			**+**
PRIC285			
RAD54L2			
TDRD12			
YTHDC2			
ZRANB3 (DNA repair?)			

*Additional characteristics that possibly are important for leukemogenesis are given in parenthesis at the end of the functional description (for additional details, see Table S3 in Supplementary Material). To the right in the figure is indicated whether the proteins are important for the function of DNA, RNA function/metabolism, or transcriptional regulation*.

## Discussion

Several recent studies have emphasized the importance of investigating the cancer cell phenotype for understanding carcinogenesis, defining patient subsets in heterogeneous malignancies, and identifying possible therapeutic targets (23–25). The aim of the present study was therefore to characterize the AML cell phenotype more in detail for the subset of patients having chemokine-responsive leukemic cells. Our present observations suggest that these patients respond to exogenous chemokines and combination of chemokine/chemokine receptor targeting either with other targeted therapies (e.g., epigenetic or integrin-targeting) or conventional chemotherapy may therefore be considered especially for the identified patient subset.

Chemokine receptor blockers can be used as an immunosuppressive strategy, e.g., as GVHD therapy in AML patients receiving allogeneic stem cell transplantation (21). Chemokine targeting may thus be used in these patients (i) as a pre-/posttransplant antileukemic strategy with direct effects on the *leukemic* cells at least for patients with chemokine-responsive cells or (ii) as an immunosuppressive treatment. GVHD has antileukemic effects, and immunosuppression due to severe GVHD will reduce this effect. However, at least for patients with chemokine-responsive AML cells, the use of chemokine targeting may represent a therapeutic alternative that reduces the GVHD-associated antileukemic reactivity but at the same time has an additional antileukemic effect by itself against residual AML.

Acute myeloid leukemia is a heterogeneous and aggressive disease, and in a previous study of another smaller patient population, we observed that exogenous CCL28 could modulate cytokine-dependent AML cell proliferation for a minority of patients. For this patient subset, CCL28 was one out of several chemokines with growth-modulating effect (4). Several chemokine receptor inhibitors are now available, and this strategy is considered in the treatment of AML both as an AML-directed antileukemic treatment or as an immunosuppressive strategy for patients with immune-mediated complications following allogeneic stem cell transplantation (21). In the present article, we have studied a large group of patients more in detail, and for these patients, the growth modulation seems to be a part of a wider phenotype that also includes altered epigenetic/transcriptional regulation, RNA metabolism/splicing, and local regulation of angiogenesis. Many chemokines and chemokine receptors show promiscuous receptor/ligand binding, and targeting of single chemokines may therefore have limited effect because a wide range of chemokines are present in the bone marrow microenvironment (9). Combined targeting with different GPCR antagonists or combination with agents that target other aspects of this complex phenotype may then be considered for this particular subset of patients.

CCL28 is a chemokine involved in normal hematopoiesis (12). We now describe that it can also be involved in leukemic hematopoiesis and modulate the growth of primary human AML cells for a subset of patients. However, its final effect depends on the local cytokine network. Growth inhibition was observed in the presence of the exogenous cytokines, SCF and especially GM-CSF, whereas growth enhancement was seen in the presence of exogenous Flt3L. Finally, CCL28 can be constitutively released by primary human AML cells and thereby be a part of the cross talk between leukemic cells and their neighboring bone marrow cells, but the ability of constitutive CCL28 release showed no association with autocrine proliferation or the effects of exogenous CCL28.

We included 79 patients in the present study, and they represent a consecutive and thereby unselected group, except that all of them showed relatively high levels of circulating AML cells. This strategy was used to allow a standardized preparation of highly enriched AML cells by gradient separation alone, thereby avoiding the risk of inducing functional alterations in the cells by more extensive separation procedures (17). The use of this strategy has been discussed previously (17), and our study population does not differ from the overall patient population regarding important biological, clinical, and prognostic parameters (4) (see below).

In our study, we investigated primary AML cells from consecutive/unselected patients. These cell populations have a hierarchical organization with (i) a majority of more mature cells undergoing stress-induced apoptosis during *in vitro* culture; (ii) an intermediate subset of clonogenic progenitors (often <1%); and (iii) a small minority of AML stem cells (18, 26). Studies of the total cell population should still be regarded as relevant despite this organization, especially with regard to antileukemic therapy, because: (i) early morphological disease control with remission induction (i.e., reduction of the total AML cell population) is an important prognostic parameter (1); (ii) clinical studies have shown that biological characteristics of the total AML cell population reflect general leukemic cell characteristics that have prognostic impact and are essential for leukemogenesis and chemosensitivity (26); and (iii) previous studies have also shown that the AML stem cells can be detected in various subsets, usually in the CD34^+^CD38^−^ subset and also in the CD34^−^ and CD34^+^CD38^+^ subsets, and stem cell characteristics are also reflected in the overall cell population (18, 26).

All our methods for cell preparation, *in vitro* culture, and AML cell characterization have been described in previous methodological investigations. First, our approach with inclusion of only patients with relatively high peripheral blood AML cell counts have been characterized previously when consecutive patients with high blood AML cell counts were compared with the overall consecutive AML patient population (17). The two groups did not differ with regard to other prognostic (i.e., chemosensitivity) parameters, but despite this, one should be careful when generalizing from our results (17). Second, the use of cryopreservation has also been discussed previously, and its effect on AML cell viability has been described in detail (19, 20). Third, we used serum-free medium for culture of AML cells (27), the only exception being the cocultures with MSCs, because these mesenchymal cells require an enriched medium (9). Finally, as described above, the AML cell population has a hierarchical organization (26), and our proliferation assay based on ^3^H-thymidine incorporation allows us to analyze only the proliferation of long-term surviving cells able to proliferate after 7 days of culture; these cells represents an enrichment of clonogenic cells (18). Thus, our study is based on well-characterized and standardized methodological strategies.

Chemokine receptor inhibitors are now being developed (28, 29). However, patients showing chemokine-dependent growth modulation usually respond to a wide range of chemokines that bind different receptors (4), and both the constitutive chemokine/cytokine release by AML cells and the release by neighboring bone marrow stromal cells will determine the final effect of chemokine receptor binding on AML cell proliferation. These observations suggest that combined targeting of promiscuous chemokine receptors would be most effective in AML. However, the intracellular signaling downstream to the various chemokine receptors is difficult to predict (16, 22), and our present results show that even such broad targeting of a promiscuous receptor has divergent effects for chemokine-responsive patients. It is not known whether combined targeting of the other phenotypic characteristics of these patients will be more effective.

Constitutive CCL28 release was observed for a minority of patients and showed no correlation with autocrine proliferation, chemokine responsiveness, or gene expression profiles. Despite these observations, CCL28 may contribute to leukemogenesis or chemosensitivity through its effect on neighboring stromal cells. Recent studies have demonstrated that CCR3 and CCR10 receptors are expressed by bone marrow stromal cells (30). The bone marrow microenvironment is hypoxic, and studies in experimental models strongly suggest that CCL28 is important for cell trafficking and angiogenesis in hypoxic microenvironments associated with carcinogenesis (31). CCL28 is also released by endothelial cells and may thereby be a part of the cross talk between AML and neighboring stromal cells. Through its release by non-leukemic stromal cells in the bone marrow microenvironment (7, 32), CCL28 may still function as a growth factor for remaining normal hematopoietic cells in AML (12). Finally, as discussed below, there may also be an interaction between integrins and CCL28, as the latter promotes integrin-dependent cell adhesion (33).

We investigated the effect of CCR1/CCR3 inhibition (both being promiscuous receptors) in a more physiological model of AML/MSC cocultures; this model was used to study chemokine effects in a local cytokine network with a major influence of bone marrow stromal elements. Our results show that even in a cytokine environment formed by the constitutive cytokine release and the cytokine-mediated cross talk between primary AML and bone marrow MSCs, the CCR1/CCR3-mediated effects on AML cell proliferation are divergent, and the different effects of CCR1/CCR3 inhibition on AML cell viability and proliferation can be explained by the hierarchical organization of the AML cell population (17). The viability studies then reflect the characteristics of the majority of more mature cells that undergo spontaneous apoptosis during the first days of culture, whereas the proliferation is assayed after several days of culture and reflects the ability of a more immature cell minority capable of maintaining proliferation after several days.

Even though we could not detect effects of chemokine inhibition on AML cell proliferation *in vitro*, studies in chronic lymphocytic leukemia (CLL) have shown that altered regulation of *in vivo* cell trafficking can contribute significantly to the antileukemic effects of certain drugs (34–39). The microenvironment is important both for survival and proliferation of CLL cells, and local chemokine release is a part of this support. Furthermore, ibrutinib mediates its antileukemic effect in CLL through direct effects on the CLL cells and through inhibition of chemokine-dependent leukemia cell trafficking, thereby keeping the cells out of their growth-enhancing/antiapoptotic microenvironment and making them susceptible to the direct antileukemic effect. Our present study shows that a subset of patients has chemokine-responsive AML cells, and similar dual-targeting may be possible in AML. This hypothesis is supported by recent studies; ibrutinib has direct antileukemic effects in human AML (40) and is in addition able to alter chemokine-dependent AML cell migration (41). Our present studies therefore suggest combined targeting of AML cell survival, and migration (i.e., chemokine targeting) should be considered for patients with chemokine-responsive AML cells.

In our studies of global gene expression profiles, we observed that AML cells with CCL28-induced growth modulation (i.e., chemokine-responsive) showed an altered expression of genes included in the GO-terms lysine acetylation/integrins/helicases. As discussed above, there is a functional interaction between CCL28 and integrins, but there is also a biological interaction between the integrin system and lysine acetylation. First, histone acetylation is important for regulation of gene expression (2, 42), including the expression of integrins in human AML cells (43). Second, lysine acetylation can also alter integrin expression indirectly; HOX transcription factors are important for regulation of integrin gene expression, and acetylation is then important in the regulation of HOX activity both through effects on the mRNA expression of these transcription factors and through modulation of their transcription factor activity by posttranscriptional modulation of HOX proteins (44, 45). Third, experimental studies suggest that integrins can mediate intracellular signaling that alters histone acetylation and thereby alter the proliferation of malignant cells (46, 47). Through this signaling, integrins can also regulate gene expression in human myeloid cells through the activation of several transcription factors (48). Finally, acetylation of microtubules is important for regulation of the cell surface density of integrins (49). Thus, through their interactions with extracellular matrix and neighboring cells, integrins mediate bidirectional signaling, i.e., inside-out and outside-in signaling (50, 51), and lysine acetylation is important at several steps of this process.

Helicases are also important for transcriptional and translational regulation and thereby seem to represent a functional overlap with lysine acetylation. First, a major part of the helicases with altered expression are members of the DEAD box family of RNA helicases, they belong to the helicase superfamily 2 together with the DEAH helicases and are often referred to as DExD/H box RNA helicases (52, 53). The helicases should be regarded as multifunctional proteins important in regulation of transcription, ribosome biogenesis, translation, and RNA decay/storage/metabolism; they also have ATPase activity and can function as metabolic sensors (53–55). The helicase DDX10 can be involved in leukemogenesis (56). Second, the mini-chromosome maintenance complex forms an important part of the replication machinery, but its loading to chromatin at sites distant from replicating DNA strongly suggests that it has additional functions, possibly as transcriptional regulators (57). Third, SWI/SNF is a multisubunit chromatin-remodeling complex important for regulation of cell cycle progression, apoptosis, differentiation, genomic instability, and DNA repair (58). The function of this molecular complex in AML seems to differ from other malignancies, and it is considered as a possible therapeutic target in human AML (59, 60).

## Conclusion

Chemokines have growth-modulating effects on the AML cells only for a minority of patients; and these patients are then responsive to several chemokines. However, this chemokine responsiveness is only a part of a more extensive leukemia cell phenotype that includes several partly interacting cellular functions, i.e., altered extracellular communication (chemokines/integrins/acetylation) and transcriptional regulation (histone acetylation, helicases) compared with the other non-responsive AML cells. Furthermore, the direct effects of chemokine targeting on primary human AML cells are difficult to predict because they depend both on differences among patients and on the overall local cytokine network; this is probably true both if chemokine targeting is tried as an antileukemic strategy or as an approach to treat immune-mediated complications in allotransplanted AML. A careful evaluation of the AML cells will possibly be important to avoid the risk of leukemia enhancement and thereby increased relapse risk; this would probably be important if the treatment is tried both as an antileukemic and an immunosuppressive strategy. A possible approach to avoid this risk may be combined targeting of other aspects of this complex phenotype. However, based on the lessons from recent studies in CLL where chemokines are important for the supportive function of neighboring stromal cells by keeping the CLL cells within their permissive microenvironment, one would emphasize that AML cells from chemokine-responsive patients should be further tested in relevant *in vivo* models (e.g., patient-derived xenografts) to see whether chemokine targeting alters AML cell trafficking/homing and thereby increases their chemosensitivity.

## Author Contributions

ØB conceived and designed the experiments; AB performed the experiments; AB and HR analyzed the data; and ØB and AB wrote the paper.

## Conflict of Interest Statement

The authors declare that the research was conducted in the absence of any commercial or financial relationships that could be construed as a potential conflict of interest.

## Abbreviations

AML, acute myeloid leukemia; CLL, chronic lymphocytic leukemia; cpm, counts per minute; ELISA, enzyme-linked immunosorbent assay; FAB, French–American–British; Flt3, FMS-like tyrosine kinase 3; Flt3L, Flt3 ligand; GM-CSF, granulocyte macrophage colony-stimulating factor; GO, gene ontology; GPCR, G-protein-coupled receptor; GVHD, graft vs. host disease; NPM1, nucleophosmin; SCF, stem cell factor; SNF/SW1, sucrose non-fermentable/SWItch.
