# Supplementary material for: A Subset of Patients with Acute Myeloid Leukemia Has Leukemia Cells Characterized by Chemokine Responsiveness and Altered Expression of Transcriptional as well as Angiogenic Regulators
Source: Front Immunol. 2016 May 25;7:205. doi: 10.3389/fimmu.2016.00205 (PMC4879142; doi:10.3389/fimmu.2016.00205)
Supplement: Supplementary file 1 [file data_sheet_1.pdf]

Supplementary Material

A subset of patients with acute myeloid leukemia has leukemia cells characterized by chemokine responsiveness and altered expression of transcriptional as well as angiogenic regulators

Annette K. Brenner*, Håkon Reikvam, Øystein Bruserud

*** Correspondence:** PhD Annette Brenner: annette.brenner@uib.no

Supplementary Tables

Supplementary Table 1 The GO term Integrins; differential gene expression when comparing AML cell with and without CCL28-induced growth modulation. The table is based on information from the GENE database and additional references given in the reference list.

GENE	PROTEIN FUNCTION	CLASSIFICATION
ADAM2	*A disintegrin and metallopeptidase domain 2.* This gene encodes a member of the ADAM (a disintegrin and metalloprotease domain) family, proteins that are membrane-anchored proteins important for cell-cell and cell-matrix interactions (1-6).	Protease
ADAM22	*ADAM metallopeptidase domain 22.* This ADAM family member is a membrane-anchored protein lacking metalloprotease activity since it has no zinc-binding motif, and it possibly functions as an integrin ligand. ADAM22 can be important for development and chemosensitivity of human malignancies (7, 8).	Protease
ADAM23	*ADAM metallopeptidase domain 23.* This protein is a member of the ADAM family of membrane-anchored proteins important for cell-cell and cell-matrix interactions. It may be involved in human carcinogenesis.	Protease
ADAMTS8	*ADAM metallopeptidase with thrombospondin type 1 motif, 8.* The protein is gene encodes a member of the ADAMTS (a disintegrin and metalloproteinase with thrombospondin motifs) family. These proteins share a propeptide region, a metalloproteinase domain, a disintegrin-like domain, and a thrombospondin type 1 (TS) motif. ADAMTS8 disrupts angiogenesis *in vivo* (9, 10).	Protease
ANGPTL1	*Angiopoietin-like 1.* Angiopoietins are vascular endothelial growth factors, and the angiopoietin/Tie2 system seems to have a prognostic impact in human AML and participates in the formation of blood vessels. This secretory protein is widely expressed in adult tissues with mRNA levels highest in highly vascularized tissues.	Angiogenesis
COL3A1	Collagen, type III, alpha 1. This protein is a fibrillar collagen that is found in several tissues, including the vascular system. The protein can be involved in cancer-associated angiogenesis (11).	Collagen
COL5A1	*Collagen, type V, alpha 1.* This gene encodes an alpha chain for one of the low abundance fibrillar collagens. Mutations in this gene are associated with Ehlers-Danlos syndrome.	Collagen
COL16A1	Collagen, type XVI, alpha 1. The alpha chain of type XVI collagen is a fibril-associated collagen that serves to maintain the integrity of the extracellular matrix.	Collagen
CYR61	Cysteine-rich, angiogenic inducer, 61. This secreted protein is growth factor-inducible and promotes the adhesion of endothelial cells. The encoded protein interacts with several integrins and with heparan sulfate proteoglycan, and it is involved in angiogenesis.	Angiogenesis
EMP2	*Epithelial membrane protein 2.* This protein regulates cell membrane composition and has been associated with endocytosis, cell signaling, cell proliferation, cell migration, cell adhesion and cell death. It is a negative regulator of caveolin-1, a scaffolding protein which is the main component of the caveolae plasma membrane invaginations. Through activation of PTK2 it positively regulates vascular endothelial growth factor A, and it also modulates the function of specific integrin isomers in the plasma membrane. Up-regulation of this gene has been linked to cancer progression in multiple different tissues.	Surface membrane
FBLN5	*Fibulin 5.* This is a secreted, extracellular matrix protein containing an Arg-Gly-Asp (RGD) motif and calcium-binding EGF-like domains. It promotes adhesion of endothelial cells through interaction of integrins and the RGD motif. Its expression is induced in injured vessels, notably in intimal vascular smooth muscle cells and endothelial cells. Therefore, the protein may play a role in vascular development and remodeling.	Extracellular matrix

GENE	PROTEIN FUNCTION	CLASSIFICATION
ICAM3	*Intercellular adhesion molecule 3.* The protein belongs to the intercellular adhesion molecule (ICAM) family; all ICAM proteins are type I transmembrane glycoproteins that bind to the LFA-1 integrin protein. LFA-1 is expressed by all leucocytes as well as endothelial cells (12).	Adhesion
IMPAD1	*Inositol monophosphatase domain containing 1.* This protein is a member of the inositol monophosphatase family, is localized to the Golgi apparatus and catalyzes the hydrolysis of phosphoadenosine phosphate to AMP. Possibly involved in carcinogenesis (13).	Golgi
ITGB1BP1	*Integrin beta 1 binding protein 1.* The encoded protein binds to the beta1 integrin cytoplasmic domain. The longer isoform form is a phosphoprotein and the extent of its phosphorylation is regulated by the cell-matrix interaction. Integrin beta 1 is important in angiogenesis (12, 14, 15).	Integrin
ITGB6	*Integrin, beta 6.* This integrin forms a dimer with an alpha v chain and this heterodimer can bind to ligands like fibronectin and transforming growth factor beta 1. This integrin seems important in angioregulation (16).	Integrin
JAM3	*Junctional adhesion molecule 3.* The protein only forms weak homotypic interactions between cells as a member of the junctional adhesion molecule protein family and acting as a receptor for another family member. It also exist in a soluble form that is important in angiogenesis (including tumor angiogenesis), and modulates VR-cadherin-mediated cell-cell contact (17-19).	Adhesion molecule<br>Angiogenesis
KDR	*Kinase* ***insert*** *domain receptor.* The gene encodes one of the two VEGF receptors; this receptor is a type III receptor tyrosine kinase. The signaling and trafficking of this receptor are regulated by multiple factors, including Rab GTPase, P2Y purine nucleotide receptor, integrin alphaVbeta3, T-cell protein tyrosine phosphatase.	VEGF receptor
LAMA5	*Laminin, alpha 5.* This laminin alpha chain is the alpha-5 subunit of laminin-10 (laminin-511), laminin-11 (laminin-521) and laminin-15 (laminin-523).	Laminin
LAMB2	*Laminin beta 2.* Laminins are noncollagenous extracellular matrix glycoproteins implicated in a wide variety of biological processes including cell adhesion, differentiation, migration, signaling, and cancer metastasis. This gene encodes the beta chain isoform laminin, beta 2. It is enriched in the basement membrane of muscles at kidney glomerulus and vascular smooth muscle. Laminins seem important in angiogenesis/blood vessel biology (20-22).	Laminin
NMB	*Neuromedin B.* This protein is a member of the bombesin-like molecule family and can bind to the neuromedin B receptor. Neuromedin B is involved in angiogenesis (23).	Angiogenesis
OXCT1	*3-oxoacid CoA transferase 1.* The protein is a mitochondrial matrix enzyme that catalyzes the reversible transfer of coenzyme A from succinyl-CoA to acetoacetate.	Metabolism
SOD1	*Superoxide dismutase 1, soluble.* The protein a cytoplasmic protein that is one of two isozymes responsible for destroying free superoxide radicals. The protein is a regulator of intracellular protein phosphorylation; inhibition of the enzyme has antiangiogenic effects (24, 25).	Signaling
THBS4	*Thrombospondin 4* is a thrombospondin family member and mediates cell-to-cell and cell-to-matrix interactions. This gene is activated during the stromal response to invasive cancer, and it is also important in the regulation of angiogenesis (26, 27).	Angiogenesis
THY1	*Thy-1 cell surface antigen.* This cell surface glycoprotein is involved in cell adhesion and cell communication, and it is used as a marker for hematopoietic stem cells. This gene may function as a tumor suppressor, and may also be important in angioregulation (28-30).	Cell membrane
TNN	*Tenascin N.* This is a protein-encoding gene. Extracellular matrix proteins of the tenascin family resemble each other in their domain structure, and also share functions in modulating cell adhesion and cellular responses to growth factors (31). These molecules may also be involved in angiogenesis (32-36).	Extracellular matrix.
VWF	*von Willebrand factor.* This is a glycoprotein involved in hemostasis. The encoded preproprotein is processed into large multimeric complexes that function in platelet adhesion and transport of various proteins in the blood. This molecule has several other functions, including modulation of angiogenesis (37, 38).	Coagulation<br>Angiogenesis

Supplementary Table 2 The GO term Histone acetylation; differential gene expression when comparing AML cell with and without CCL28-induced growth modulation. The table is based on information from the GENE database and additional references given in the reference list.

GENE	PROTEIN FUNCTION	CLASSIFICATION
ACTL6A	*Actin-like 6A.* The protein is a family member of actin-related proteins (ARPs); it is a 53 kDa subunit protein of the BAF (BRG1/brm-associated factor) complex thought to facilitate transcriptional activation of specific genes by antagonizing chromatin-mediated transcriptional repression.	Chromatin modulation
BAT3	*BCL2-associated athanogene 6.* This gene encodes a nuclear protein implicated in the control of apoptosis. In addition, the protein forms a complex with E1A binding protein p300 and is required for p53 acetylation in response to DNA damage.	Apoptosis
BRCA2	*Breast cancer 2, early onset.* Both BRCA1 and BRCA2 are involved in maintenance of genome stability, specifically the homologous recombination pathway for double-strand DNA repair. BRCA2 is considered a tumor suppressor gene.	DNA repair
BRD8	*Bromodomain containing 8.* The protein contains a bromodomain and is thought to be a nuclear receptor coactivator.	Transcription
BRPF1	*Bromodomain and PHD finger containing, 1.* The protein is localized within nuclei and is possibly a transcriptional regulator.	Transcription?
CCDC101	*Coiled-coil domain containing 101.*	
CHD9	*Chromodomain helicase DNA binding protein 9.* The Chromatin-Related Mesenchymal Modulator CHD9 is important for the nucleosomes and binds with modified H3-(K9me2/3 and K27me3); it also has a role with RNA Polymerase II (Pol II)-dependent transcription.	Chromatin modulation<br>Transcription
CPA3	Carboxypeptidase A3. This is a zinc metalloproteases that is released extracellularly and may be involved in protein degradation.	Protease
DMAP1	DNA methyltransferase 1 associated protein 1. This protein is a subunit of several, distinct complexes involved in the repression or activation of transcription. It is targeted to replication foci throughout S phase by interacting directly with the N-terminus of DNA methyltransferase 1. During late S phase, histone deacetylase 2 is added to this complex. It is also a component of the nucleosome acetyltransferase of H4 complex.	Transcription<br>Histone modification
EPC1	Enhancer of polycomb homolog 1. The protein is a component of the NuA4 histone acetyltransferase complex and can act as both a transcriptional activator and repressor.	Transcription<br>Histone modification
EP400	E1A binding protein p400 (39).	Transcription
GTF3C4	*General transcription factor IIIC, polypeptide 4, 90kDa.*	
HCFC1	*Host cell factor C1.* This gene is a member of the host cell factor family; the protein is involved in control of the cell cycle and transcriptional regulation.	Transcription<br>Cell cycle regulation
ING3	Inhibitor of growth family, member 3. This is a tumor suppressor protein that interacts with TP53, inhibit cell growth, and induce apoptosis. It contains a PHD-finger, a common motif in proteins involved in chromatin remodeling.	Tumor suppressor<br>Chromatin modulation
KAT2A	*K(lysine) acetyltransferase 2A.* KAT2A is a histone acetyltransferase (HAT) that functions primarily as a transcriptional activator. It also functions as a repressor of NF-kappa-B (see MIM 164011) by promoting ubiquitination of the NF-kappa-B subunit RELA (MIM 164014) in a HAT-independent manner	Transcription
KAT2B	K(lysine) acetyltransferase 2B. The protein encoded by this gene associates with p300/CBP. It has histone acetyl transferase activity with core histones and nucleosome core particles.	Transcription<br>Histone modulation

GENE	PROTEIN FUNCTION	CLASSIFICATION
KIAA1267	KAT8 regulatory NSL complex subunit 1. This is a nuclear protein that is a subunit of two protein complexes involved with histone acetylation, the MLL1 complex and the NSL1 complex.	Histone acetylation
KIAA1310	*KAT8 regulatory NSL complex subunit 3.*	
LDB1	LIM domain binding 1. A known transcriptional regulator in hematopoiesis (40).	Transcription
MBIP	*AP3K12 binding inhibitory protein.*	
MEAF6	*MYST/Esa1-associated factor 6.* This nuclear protein is involved in transcriptional activation. The encoded protein may form a component of several different histone acetyltransferase complexes.	Histone acetylation<br>Transcription
MECP2	*Methyl CpG binding protein 2.* MECP2 has a methyl-CpG binding domain and can bind specifically to methylated DNA; it can repress transcription from methylated gene promoters.	Transcription
MLL	Lysine (K)-specific methyltransferase 2A. This transcriptional coactivator plays an essential role in regulating gene expression during hematopoiesis. It has histone H3 lysine 4 (H3K4) methyltransferase activity. This protein is processed into the MLL-C and MLL-N fragments that reassociate and further assemble into different multiprotein complexes that regulate the transcription of specific target genes, including many of the HOX genes.	Transcription<br>Histone modification
MSL2	*Male-specific lethal 2 homolog.*	
MYOD1	Myogenic differentiation 1. This nuclear protein is a member of the basic helix-loop-helix family of transcription factors.	Transcription
MYST1	*Probable histone acetyltransferase MYST1-like.* This gene encodes a member of the MYST histone acetylase protein family.	Transcription<br>Histone acetylation
MYST2	*MYST histone acetyltransferase 2*	Transcription?<br>Histone acetylation?
MYST4	*K(lysine) acetyltransferase 6B.*	Transcription?<br>Histone acetylation?
OGT	O-linked N-acetylglucosamine (GlcNAc) transferase. This glycosyltransferase catalyzes the addition of a single N-acetylglucosamine in O-glycosidic linkage to serine or threonine residues. It functions as a transcriptional regulator (41-43).	Transcription
PHF15	*PHD finger protein 15.*	
PHF16	*PHD finger protein 16.*	
PHF17	PHD finger protein 17.	Transcription?
PHF20	*PHD finger protein 20.* The protein functions as a tumor suppressor and is important for modulation of NFκB signaling, DNA damage and the function of p53 (44-46).	Transcription<br>DNA repair
SMARCA4	*SWI/SNF related, matrix associated, actin dependent regulator of chromatin, subfamily a, member 4.* The protein is thought to regulate transcription of certain genes by altering the chromatin structure around those genes. It is part of the large ATP-dependent chromatin remodeling complex SNF/SWI, which is required for transcriptional activation of genes normally repressed by chromatin.	Transcription<br>Chromatin modulation
SRCAP	Snf2-related CREBBP activator protein. This protein is the core catalytic component of the multiprotein chromatin-remodeling SRCAP complex. It is necessary for incorporation of the histone variant H2A.Z into nucleosomes and can function as a transcriptional activator in Notch-mediated, CREB-mediated and steroid receptor-mediated transcription.	Transcription<br>Histone-chromatin modulation

GENE	PROTEIN FUNCTION	CLASSIFICATION
TAF1	*TAF1 RNA polymerase II, TATA box binding protein (TBP)-associated factor.* Initiation of transcription by RNA polymerase II requires the activities of more than 70 polypeptides coordinated by the basal transcription factor TFIID. TFIID is composed of the TATA-binding protein (TBP) and a group of evolutionarily conserved proteins known as TBP-associated factors or TAFs. TAFs may participate in basal transcription, serve as coactivators, function in promoter recognition or modify general transcription factors (GTFs) to facilitate complex assembly and transcription initiation. This gene encodes the largest subunit of TFIID, but it also binds to other transcriptional regulators, possesses acetyltransferase activity and can act as an ubiquitin-activating/conjugating enzyme.	Transcription
TAF1L	TAF1 RNA polymerase II, TATA box binding protein (TBP)-associated factor, 210kDa-like. This locus is intronless, and the product has been shown to function interchangeably with the TAF1 product.	Transcription
TAF6L	TAF6-like RNA polymerase II, p300/CBP-associated factor (PCAF)-associated factor. Initiation of transcription by RNA polymerase II requires the activities of more than 70 polypeptides coordinated by transcription factor IID (TFIID) TFIID is composed of the TATA-binding protein (TBP) and a group of proteins known as TBP-associated factors or TAFs. The protein is a component of the PCAF histone acetylase complex and structurally similar to TAF6.	Transcription<br>Histone modification
TAF15	TAF15 RNA polymerase II, TATA box binding protein (TBP)-associated factor. This protein is a member of the TET family of RNA-binding proteins and is involved in RNA polymerase II gene transcription as a component of the TFIID complexes.	Transcription
TCF3	*Transcription factor 3.* The protein is a helix-loop-helix transcription factor. This gene is involved in acute leukemia t(12;19) together with ZNF384).	Transcription
TRRAP	*Transformation/transcription domain-associated protein.* This large multidomain protein of the phosphoinositide 3-kinase-related kinases (PIKK) is a common component of many histone acetyltransferase (HAT) complexes and plays a role in transcription and DNA repair by recruiting HAT complexes to chromatin.	Histone acetylation<br>DNA repair<br>Transcription
USP22	*Ubiquitin specific peptidase 22.*	
YEATS2	*YEATS domain containing 2.* YEATS2 is a scaffolding subunit of the ADA2A (TADA2A; MIM 602276)-containing (ATAC) histone acetyltransferase complex.	Transcription

Supplementary Table 3 The GO term Helicase Activity; differential gene expression when comparing AML cell with and without CCL28-induced growth modulation. The table is based on information from the GENE database.

GENE	PROTEIN FUNCTION	CLASSIFICATION
ASCC3	*Activating signal cointegrator 1 complex subunit 3.* This protein belongs to a family of helicases that are involved in the ATP-dependent unwinding of nucleic acid duplexes. The encoded protein is the largest subunit of the activating signal cointegrator 1 complex that is involved in DNA repair and resistance to alkylation damage.	DNA repair<br>Chemoresistance
ATRX	*Alpha thalassemia/mental retardation syndrome X-linked.* The protein contains an ATPase/helicase domain and belongs to the SWI/SNF family of chromatin remodeling proteins. Its nuclear matrix and chromatin association is regulated by cell cycle dependent phosphorylation. The protein seems to be important both for DNA methylation, chromatin remodeling and gene expression	Chromatin remodeling<br>DNA methylation<br>Cell cycle regulation?
CHD3	*Chromodomain helicase DNA binding protein 3.* This protein is one of the components of a histone deacetylase complex referred to as the Mi-2/NuRD complex which participates in the remodeling of chromatin.	Transcription<br>Histone deacetylation
CHD4	*Chromodomain helicase DNA binding protein 4.* The protein belongs to the SNF2/RAD54 helicase family. It represents the main component of the nucleosome remodeling and deacetylase complex and plays an important role in epigenetic transcriptional repression.	Epigenetic regulation
CHD6	*Chromodomain helicase DNA binding protein 6.* The protein is a member of the SNF2/RAD54 helicase family. The encoded protein contains two chromodomains, a helicase domain, and an ATPase domain. It is thought to be a core member of one or more of the chromatin remodeling complexes. The encoded protein may function as a transcriptional repressor.	SNF2/RAD54 helicase family<br>Chromatin remodeling and regulation of transcription
CHD8	*Chromodomain helicase DNA binding protein 8.* This DNA helicase functions as a transcription repressor by remodeling chromatin structure. It binds beta-catenin and negatively regulates Wnt signaling pathway.	Transcription<br>Chromatin remodeling
CHD9	Chromodomain helicase DNA binding.	
DDX3X	*DEAD (Asp-Glu-Ala-Asp) box helicase 3, X-linked.* The protein is a member of the DEAD-box protein family. This protein has a high level of RNA-independent ATPase activity, and unlike most DEAD-box helicases, the ATPase activity is thought to be stimulated by both RNA and DNA. It is thought to play roles in both the nucleus and cytoplasm. Nuclear roles include transcriptional regulation, mRNP assembly, pre-mRNA splicing, and mRNA export. In the cytoplasm, this protein is thought to be involved in translation, cellular signaling, and viral replication. Misregulation of this gene has been implicated in tumorigenesis.	DEAD-box family<br>Transcription, translation, signaling
DDX4	*DEAD (Asp-Glu-Ala-Asp) box polypeptide 4.* This is a DEAD box proteins/helicase family member, it is an RNA helicase.	DEAD box family member
DDX6	*DEAD (Asp-Glu-Ala-Asp) box helicase 6.* The protein is a DEAD box family member. It is an RNA helicase found in P-bodies and stress granules, and functions in translation suppression and mRNA degradation. It is required for microRNA-induced gene silencing.	DEAD box family<br>Transcription
DDX10	*DEAD (Asp-Glu-Ala-Asp) box polypeptide 10.* This DEAD box protein/RNA helicase that may be involved in ribosome assembly. Fusion of this gene and the nucleoporin gene, NUP98, by inversion 11 (p15q22) chromosome translocation is found in the patients with de novo or therapy-related myeloid malignancies	DEAD box protein<br>Leukemogenesis?
DDX17	*DEAD (Asp-Glu-Ala-Asp) box helicase 17.* This RNA DEAD box protein/helicase is an ATPase activated by a variety of RNA species, but not by dsDNA.	DEAD box family
DDX18	*DEAD (Asp-Glu-Ala-Asp) box polypeptide 18.* This DEAD box protein/RNA helicase is activated by Myc protein.	DEAD box protein

GENE	PROTEIN FUNCTION	CLASSIFICATION
DDX27	*DEAD (Asp-Glu-Ala-Asp) box polypeptide 27.* This is a DEAD box protein/helicase with unknown function.	DEAD box protein
DDX28	*DEAD (Asp-Glu-Ala-Asp) box polypeptide 28.* This is an RNA helicase, being a member of the DEAD box protein family. The encoded protein is localized in the mitochondria and the nucleus.	DEAD box family<br>Transcription?
DDX42	*DEAD (Asp-Glu-Ala-Asp) box helicase 42.* This DEAD box protein/RNA helicase.	DEAD box family
DDX46	*DEAD (Asp-Glu-Ala-Asp) box polypeptide 46.* This member of the DEAD box protein/RNA helicase family is a component of the 17S U2 snRNP complex; it plays an important role in pre-mRNA splicing.	DEAD box family<br>RNA splicing
DDX50	*DEAD (Asp-Glu-Ala-Asp) box polypeptide 50.* This is a DEAD box protein/helicase family member that may be involved in ribosomal RNA synthesis or processing. This gene and DDX21 have similar genomic structures	DEAD box family<br>RNA synthesis
DDX51	DEAD (Asp-Glu-Ala-Asp) box polypeptide 51.	DEAD box family
DDX52	DEAD (Asp-Glu-Ala-Asp) box polypeptide 52.	DEAD box
DDX54	*DEAD (Asp-Glu-Ala-Asp) box polypeptide 54.* The protein is a DEAD box family member, it is a nucleolar protein interacts in a hormone-dependent manner with nuclear receptors and represses their transcriptional activity.	DEAD-box family<br>Transcriptional regulation
DDX58	*DEAD (Asp-Glu-Ala-Asp) box polypeptide 58.* This member of the DEAD box protein family of RNA helicases has a caspase recruitment domain (CARD).	DEAD box family
DDX60	*DEAD (Asp-Glu-Ala-Asp) box polypeptide 60.*	DEAD box family
DDX60L	*DEAD (Asp-Glu-Ala-Asp) box polypeptide 60-like*	DEAD family
DHX8	*DEAH (Asp-Glu-Ala-His) box polypeptide 8.* This is a member of the DEAH box polypeptide family is thought to function as an ATP-dependent RNA helicase that regulates the release of spliced mRNAs from spliceosomes prior to their export from the nucleus.	DEAH box family
DHX9	*DEAH (Asp-Glu-Ala-His) box helicase 9.* This protein is a member of the DEAH-containing family of RNA helicases; it is an enzyme that catalyzes the ATP-dependent unwinding of double-stranded RNA and DNA-RNA complexes. This protein localizes to both the nucleus and the cytoplasm and functions as a transcriptional regulator.	Transcriptional regulation<br>DEAH family member
DHX16	*DEAH (Asp-Glu-Ala-His) box polypeptide 16.* This DEAD box protein/RNA helicase is a functional homolog of fission yeast Prp8 protein involved in cell cycle progression.	DEAD box family
DHX29	*DEAH (Asp-Glu-Ala-His) box polypeptide 29.*	DEAH box family
DHX30	*DEAH (Asp-Glu-Ala-His) box helicase 30.* The protein is a DEAD box protein/RNA helicase. It is a mitochondrial nucleoid protein associated with mitochondrial DNA. It has also been identified as a component of a transcriptional repressor complex	DEAD box family
DHX32	*DEAH (Asp-Glu-Ala-His) box polypeptide 32.* DEAD box proteins are RNA helicases and characterized by the conserved motif Asp-Glu-Ala-Asp (DEAD). They are implicated in alteration of the RNA secondary structure and thereby in translation initiation, nuclear and mitochondrial splicing as well as ribosome and spliceosome assembly. Some of these proteins are believed to be involved in regulation of cell growth/division. The function of this member is unknown.	DEAD box protein
DHX33	*DEAH (Asp-Glu-Ala-His) box polypeptide 33.* This member of the DEAD box protein family. It is an RNA helicase.	DEAD box family
DHX35	*DEAH (Asp-Glu-Ala-His) box polypeptide 35.* This is a DEAD box RNA helicase; its function has not been determined.	

GENE	PROTEIN FUNCTION	CLASSIFICATION
DHX37	*DEAH (Asp-Glu-Ala-His) box polypeptide 37.* This gene encodes a DEAD box protein/RNA helicase	DEAD box helicase
DHX40	*DEAH (Asp-Glu-Ala-His) box polypeptide 40.* This protein is a member of the DExH/D box family of ATP-dependent RNA helicases that have an essential role in RNA metabolism.	DExH/D box family<br>RNA metabolism
DOX19A	DEAD (Asp-Glu-Ala-Asp) box polypeptide 19a.	
EIF4A1	Eukaryotic translation initiation factor 4A1.	
EP400	*E1A binding protein p400.*	
ERCC8	*Excision repair cross-complementation group 8.* This protein interacts with the Cockayne syndrome type B (CSB) protein and the p44 protein, a subunit of the RNA polymerase II transcription factor IIH. Involved in the repair of transcriptionally active genes.	Transcription
GTF2F2	General transcription factor IIF, polypeptide 2, 30kDa.	Transcription
HELZ	*Helicase with zinc finger.* HELZ is a member of the superfamily I class of RNA helicases that alter the conformation of RNA by unwinding double-stranded regions, thereby altering the biologic activity of RNA and regulating access to other proteins.	RNA helicase
HLTF	*Helicase-like transcription factor.* The protein is a member of the SWI/SNF family and contains a RING finger DNA binding motif.	SWI/SNF family<br>Transcription?
IFIH1	*Interferon induced with helicase C domain.* This gene encodes a DEAD box protein that is upregulated in response to treatment with beta-interferon and protein kinase C-activation.	Dead box family
JARID2	*Jumonji, AT rich interactive domain 2.* The protein is a Jumonji- and AT-rich interaction domain (ARID)-domain-containing protein. It is a DNA-binding protein that functions as a transcriptional repressor, interacts with the Polycomb repressive complex 2 and facilitates the recruitment of this complex to target genes. Mutations in this gene are associated with chronic myeloid malignancies.	Transcription<br>Myeloid malignancies
MCM7	*Minichromosome maintenance complex component 7.* This is one of the highly conserved mini-chromosome maintenance proteins (MCM) that are essential for the initiation of eukaryotic genome replication. The hexameric protein complex formed by the MCM proteins is a key component of the pre-replication complex (pre-RC) and may be involved in the formation of replication forks and the recruitment of other DNA replication related proteins. The MCM complex consisting of this protein and MCM2, 4 and 6 proteins possesses DNA helicase activity and may act as a DNA unwinding enzyme. Cyclin D1-dependent kinase, CDK4, associates with this protein, and may regulate the binding of this protein with the tumorsuppressor protein RB1/RB.	Genome replication
MCM8	*Minichromosome maintenance 8 homologous recombination repair factor.* This is one of the mini-chromosome maintenance proteins (MCM) that are essential for the initiation of eukaryotic genome replication. The hexameric protein complex formed by the mini-chromosome maintenance proteins is a key component of the pre-replication complex. It may interact with other mini-chromosome maintenance proteins and play a role in DNA replication.	MCM protein<br>DNA replication
MCM9	*Minichromosome maintenance 9 homologous recombination repair factor.* The protein is a member of the mini-chromosome maintenance (MCM) protein family that are essential for the initiation of eukaryotic genome replication. Binding of this protein to chromatin is needed for recruiting the MCM2-7 helicase to DNA replication origins. This protein binds and is a positive regulator of the chromatin licensing and DNA replication factor 1, CDT1.	Genome replication

GENE	PROTEIN FUNCTION	CLASSIFICATION
MCMDC1	*Minichromosome maintenance 9 homologous recombination repair factor.* The protein is a member of the mini-chromosome maintenance (MCM) family that are essential for the initiation of eukaryotic genome replication. Binding of this protein to chromatin has been shown to be a pre-requisite for recruiting the MCM2-7 helicase to DNA replication origins. This protein also binds, and is a positive regulator of, the chromatin licensing and DNA replication factor 1, CDT1.	MCM member<br>DNA replication<br>Chromatin modulation
MOV10	*Mov10 RISC complex RNA helicase.*	
PRIC285	*Peroxisomal proliferator-activated receptor A-interacting complex 285 kDa protein-like.*	
RAD54L2	*RAD54 like 2.*	
SHPRH	*SNF2 histone linker PHD RING helicase, E3 ubiquitin protein ligase.* This protein contains motifs that are characteristic of several DNA repair proteins, transcription factors, and helicases.	DNA repair?<br>Transcription?
SMARCA2	*SWI/SNF related, matrix associated, actin dependent regulator of chromatin, subfamily a, member 2.* The protein is a member of the SWI/SNF helicase family that is thought to regulate gene transcription by altering the chromatin structure. The protein is part of the ATP-dependent chromatin remodeling complex SNF/SWI, which is required for transcriptional activation of genes normally repressed by chromatin.	SWI/SNF family<br>Chromatin remodeling<br>Transcription
SMARCA4	*SWI/SNF related, matrix associated, actin dependent regulator of chromatin, subfamily a, member 4.* The protein is a SWI/SNF family member; they that have helicase and ATPase activities and are thought to regulate transcription of certain genes by altering the chromatin structure. The encoded protein is part of the large ATP-dependent chromatin remodeling complex SNF/SWI, which is required for transcriptional activation of genes normally repressed by chromatin. The protein can bind BRCA1, as well as regulate the expression of the tumorigenic protein CD44.	Chromatin remodeling<br>SNF/SWI member
SMARCAL1	*SWI/SNF related, matrix associated, actin dependent regulator of chromatin, subfamily a-like 1.* The protein is a SWI/SNF family member.	Chromatin remodeling<br>SNF/SWI member
SRCAP	*Snf2-related CREBBP activator protein.* This gene encodes the core catalytic component of the multiprotein chromatin-remodeling SRCAP complex. The encoded protein is an ATPase that is necessary for the incorporation of the histone variant H2A.Z into nucleosomes. It can function as a transcriptional activator in Notch-, CREB- and steroid receptor-mediated transcription.	Chromatin remodeling
TDRD12	*Tudor domain containing 12.*	
WRN	*Werner syndrome, RecQ helicase-like.* This protein is a member of the RecQ subfamily and the DEAH (Asp-Glu-Ala-His) subfamily of DNA and RNA helicases. The protein shows a predominant nucleolar localization. It possesses an intrinsic 3' to 5' DNA helicase activity, and is also a 3' to 5' exonuclease. Based on interactions between this protein and Ku70/80 heterodimer in DNA end processing, this protein may be involved in the repair of double strand DNA breaks.	DEAH family<br>DNA repair
YTHDC2	*YTH domain containing 2.*	
ZRANB3	*Zinc finger, RAN-binding domain containing 3.*	

References

1. Handsley MM, Edwards DR. Metalloproteinases and their inhibitors in tumor angiogenesis. *Int J Cancer* (2005) **115**: 849-60.
2. Lafleur MA, Handsley MM, Edwards DR. Metalloproteinases and their inhibitors in angiogenesis. *Expert Rev Mol Med* (2003) **5**: 1-39.
3. Reiss K, Saftig P. The "a disintegrin and metalloprotease" (ADAM) family of sheddases: physiological and cellular functions. *Semin Cell Dev Biol* (2009) **20**: 126-37.
4. Sun Y, Huang J, Yang Z. The roles of ADAMTS in angiogenesis and cancer. *Tumour Biol* (2015) **36**: 4039-51.
5. van der Vorst EP, Keijbeck AA, de Winther MP, Donners MM. A disintegrin and metalloproteases: molecular scissors in angiogenesis, inflammation and atherosclerosis. *Atherosclerosis* (2012) **224**: 302-8.
6. van Hinsbergh VW, Engelse MA, Quax PH. Pericellular proteases in angiogenesis and vasculogenesis. *Arterioscler Thromb Vasc Biol* (2006) **26**: 716-28.
7. Bolger JC, Young LS. ADAM22 as a prognostic and therapeutic drug target in the treatment of endocrine-resistant breast cancer. *Vitam Horm* (2013) **93**: 307-21.
8. McCartan D, Bolger JC, Fagan A, Byrne C, Hao Y, Qin L, et al. Global characterization of the SRC-1 transcriptome identifies ADAM22 as an ER-independent mediator of endocrine-resistant breast cancer. *Cancer Res* (2012) **72**: 220-9.
9. Apte SS. A disintegrin-like and metalloprotease (reprolysin-type) with thrombospondin type 1 motif (ADAMTS) superfamily: functions and mechanisms. *J Biol Chem* (2009) **284**: 31493-7.
10. Wagstaff L, Kelwick R, Decock J, Edwards DR. The roles of ADAMTS metalloproteinases in tumorigenesis and metastasis. *Front Biosci (Landmark Ed)* (2011) **16**: 1861-72.
11. Liu Y, Carson-Walter EB, Cooper A, Winans BN, Johnson MD, Walter KA. Vascular gene expression patterns are conserved in primary and metastatic brain tumors. *J Neurooncol* (2010) **99**: 13-24.
12. Zimmerman T, Blanco FJ. Inhibitors targeting the LFA-1/ICAM-1 cell-adhesion interaction: design and mechanism of action. *Curr Pharm Des* (2008) **14**: 2128-39.
13. Parris TZ, Kovacs A, Hajizadeh S, Nemes S, Semaan M, Levin M, et al. Frequent MYC coamplification and DNA hypomethylation of multiple genes on 8q in 8p11-p12-amplified breast carcinomas. *Oncogenesis* (2014) **3**: e95.
14. Mettouchi A, Meneguzzi G. Distinct roles of beta1 integrins during angiogenesis. *Eur J Cell Biol* (2006) **85**: 243-7.
15. Verkarre V, Patey-Mariaud de Serre N, Vazeux R, Teillac-Hamel D, Chretien-Marquet B, Le Bihan C, et al. ICAM-3 and E-selectin endothelial cell expression differentiate two phases of angiogenesis in infantile hemangiomas. *J Cutan Pathol* (1999) **26**: 17-24.
16. Mash DC, ffrench-Mullen J, Adi N, Qin Y, Buck A, Pablo J. Gene expression in human hippocampus from cocaine abusers identifies genes which regulate extracellular matrix remodeling. *PLoS One* (2007) **2**: e1187.

17. Lamagna C, Hodivala-Dilke KM, Imhof BA, Aurrand-Lions M. Antibody against junctional adhesion molecule-C inhibits angiogenesis and tumor growth. *Cancer Res* (2005) **65**: 5703-10.

18. Orlova VV, Economopoulou M, Lupu F, Santoso S, Chavakis T. Junctional adhesion molecule-C regulates vascular endothelial permeability by modulating VE-cadherin-mediated cell-cell contacts. *J Exp Med* (2006) **203**: 2703-14.

19. Rabquer BJ, Amin MA, Teegala N, Shaheen MK, Tsou PS, Ruth JH, et al. Junctional adhesion molecule-C is a soluble mediator of angiogenesis. *J Immunol* (2010) **185**: 1777-85.

20. Dakouane-Giudicelli M, Brouillet S, Traboulsi W, Torre A, Vallat G, Si Nacer S, et al. Inhibition of human placental endothelial cell proliferation and angiogenesis by netrin-4. *Placenta* (2015) **36**: 1260-5.

21. Du R, Wang Y, Teng JF, Lu H, Lu R, Shi Z. Effect of integrin combined laminin on peripheral blood vessel of cerebral infarction and endogenous nerve regeneration. *J Biol Regul Homeost Agents* (2015) **29**: 167-74.

22. Shan N, Zhang X, Xiao X, Zhang H, Tong C, Luo X, et al. Laminin alpha4 (LAMA4) expression promotes trophoblast cell invasion, migration, and angiogenesis, and is lowered in preeclamptic placentas. *Placenta* (2015) **36**: 809-20.

23. Park HJ, Kim SR, Bae SK, Choi YK, Bae YH, Kim EC, et al. Neuromedin B induces angiogenesis via activation of ERK and Akt in endothelial cells. *Exp Cell Res* (2009) **315**: 3359-69.

24. Donate F, Juarez JC, Burnett ME, Manuia MM, Guan X, Shaw DE, et al. Identification of biomarkers for the antiangiogenic and antitumour activity of the superoxide dismutase 1 (SOD1) inhibitor tetrathiomolybdate (ATN-224). *Br J Cancer* (2008) **98**: 776-83.

25. Juarez JC, Manuia M, Burnett ME, Betancourt O, Boivin B, Shaw DE, et al. Superoxide dismutase 1 (SOD1) is essential for H2O2-mediated oxidation and inactivation of phosphatases in growth factor signaling. *Proc Natl Acad Sci U S A* (2008) **105**: 7147-52.

26. Carpizo D, Iruela-Arispe ML. Endogenous regulators of angiogenesis--emphasis on proteins with thrombospondin--type I motifs. *Cancer Metastasis Rev* (2000) **19**: 159-65.

27. Muppala S, Frolova E, Xiao R, Krukovets I, Yoon S, Hoppe G, et al. Proangiogenic Properties of Thrombospondin-4. *Arterioscler Thromb Vasc Biol* (2015) **35**: 1975-86.

28. Cina DP, Xu H, Liu L, Farkas L, Farkas D, Kolb M, et al. Renal tubular angiogenic dysregulation in anti-Thy1.1 glomerulonephritis. *Am J Physiol Renal Physiol* (2011) **300**: F488-98.

29. Tyczewska M, Rucinski M, Trejter M, Ziolkowska A, Szyszka M, Malendowicz LK. Angiogenesis in the course of enucleation-induced adrenal regeneration--expression of selected genes and proteins involved in development of capillaries. *Peptides* (2012) **38**: 404-13.

30. Wnuk M, Hlushchuk R, Tuffin G, Huynh-Do U, Djonov V. The effects of PTK787/ZK222584, an inhibitor of VEGFR and PDGFRbeta pathways, on intussusceptive

angiogenesis and glomerular recovery from Thy1.1 nephritis. *Am J Pathol* (2011) **178**: 1899-912.

31. Chiovaro F, Chiquet-Ehrismann R, Chiquet M. Transcriptional regulation of tenascin genes. *Cell Adh Migr* (2015) **9**: 34-47.

32. Behrem S, Zarkovic K, Eskinja N, Jonjic N. Distribution pattern of tenascin-C in glioblastoma: correlation with angiogenesis and tumor cell proliferation. *Pathol Oncol Res* (2005) **11**: 229-35.

33. Martina E, Degen M, Ruegg C, Merlo A, Lino MM, Chiquet-Ehrismann R, et al. Tenascin-W is a specific marker of glioma-associated blood vessels and stimulates angiogenesis in vitro. *FASEB J* (2010) **24**: 778-87.

34. Midwood KS, Hussenet T, Langlois B, Orend G. Advances in tenascin-C biology. *Cell Mol Life Sci* (2011) **68**: 3175-99.

35. Orend G, Chiquet-Ehrismann R. Tenascin-C induced signaling in cancer. *Cancer Lett* (2006) **244**: 143-63.

36. Saito Y, Shiota Y, Nishisaka M, Owaki T, Shimamura M, Fukai F. Inhibition of angiogenesis by a tenascin-c peptide which is capable of activating beta1-integrins. *Biol Pharm Bull* (2008) **31**: 1003-7.

37. Franchini M, Frattini F, Crestani S, Bonfanti C, Lippi G. von Willebrand factor and cancer: a renewed interest. *Thromb Res* (2013) **131**: 290-2.

38. Luo GP, Ni B, Yang X, Wu YZ. von Willebrand factor: more than a regulator of hemostasis and thrombosis. *Acta Haematol* (2012) **128**: 158-69.

39. Smith JA, White EA, Sowa ME, Powell ML, Ottinger M, Harper JW, et al. Genome-wide siRNA screen identifies SMCX, EP400, and Brd4 as E2-dependent regulators of human papillomavirus oncogene expression. *Proc Natl Acad Sci U S A* (2010) **107**: 3752-7.

40. Love PE, Warzecha C, Li L. Ldb1 complexes: the new master regulators of erythroid gene transcription. *Trends Genet* (2014) **30**: 1-9.

41. Lewis BA, Hanover JA. O-GlcNAc and the epigenetic regulation of gene expression. *J Biol Chem* (2014) **289**: 34440-8.

42. Ozcan S, Andrali SS, Cantrell JE. Modulation of transcription factor function by O-GlcNAc modification. *Biochim Biophys Acta* (2010) **1799**: 353-64.

43. Ruan HB, Singh JP, Li MD, Wu J, Yang X. Cracking the O-GlcNAc code in metabolism. *Trends Endocrinol Metab* (2013) **24**: 301-9.

44. Cui G, Park S, Badeaux AI, Kim D, Lee J, Thompson JR, et al. PHF20 is an effector protein of p53 double lysine methylation that stabilizes and activates p53. *Nat Struct Mol Biol* (2012) **19**: 916-24.

45. Li Y, Park J, Piao L, Kong G, Kim Y, Park KA, et al. PKB-mediated PHF20 phosphorylation on Ser291 is required for p53 function in DNA damage. *Cell Signal* (2013) **25**: 74-84.

46. Zhang T, Park KA, Li Y, Byun HS, Jeon J, Lee Y, et al. PHF20 regulates NF-kappaB signalling by disrupting recruitment of PP2A to p65. *Nat Commun* (2013) **4**: 2062.
